# Supplementary material for: Halogen hydrogen-bonded organic framework (XHOF) constructed by singlet open-shell diradical for efficient photoreduction of U(VI)
Source: Nat Commun. 2022 Mar 16;13:1389. doi: 10.1038/s41467-022-29107-9 (PMC8927584; doi:10.1038/s41467-022-29107-9)
Supplement: Supplementary file 1 — Supplementary Information [file 41467_2022_29107_MOESM1_ESM.pdf]

Supplementary Information

Halogen hydrogen-bonded organic framework (XHOF) constructed by singlet open-shell diradical for efficient photoreduction of U(VI)

Lijuan Feng, Yihui Yuan,* Bingjie Yan, Tiantian Feng, Yaping Jian, Jiacheng Zhang, Wenyan Sun, Ke Lin, Guangsheng Luo, and Ning Wang*

Contents

Supplementary Figure 1 The FTIR spectra of TPN and TPAO……………………….…………...3

Supplementary Figure 2 The MS spectrum of XHOF-TAQ…………………..…..……..……..…..4

Supplementary Figure 3 The XPS spectra of CuCl2 and XHOF-TAQ……………………….....….5

Supplementary Figure 4 The FTIR spectrum of XHOF-TAQ……………………………….……….6

Supplementary Figure 5 The PXRD patterns of XHOF-TAQ in different solvents….………...…7

Supplementary Figure 6 PXRD pattern of the as-synthesized XHOF-TAQ and the XHOF-TAQ after being placed in air for one year…...…..……..……..…..….……..……..………..…..………..….8

Supplementary Figure 7 The XPS spectra of before and after the reaction of uranium.………..….9

Supplementary Figure 8 PXRD pattern of XHOF-TAQ after being used for uranium photoreduction.……......……...……...……...……...……...……...……...……...……...…...……10

Supplementary Figure 9 The variable temperature EPR of the XHOF-TAQ.………………..…...11

Supplementary Figure 10 Temperature dependence of χ and χM curve for the powder XHOF-TAQ ……………………………………………………………………….……...……….12

Supplementary Figure 11 The DRS spectrum of the XHOF-TAQ.…….……….…….…….…....13

Supplementary Figure 12 The DRS spectrum was converted to the TAUC plot……………..…..14

Supplementary Table 1 Comparison of uranium photoreduction performance with other available materials..………..……………………………………………………….……..……15

Supplementary Table 2 The crystallographic data for XHOF-TAQ.………..…..…….……..……17

Supplementary References..………..…………………………………………………………..18

Supplementary Figures

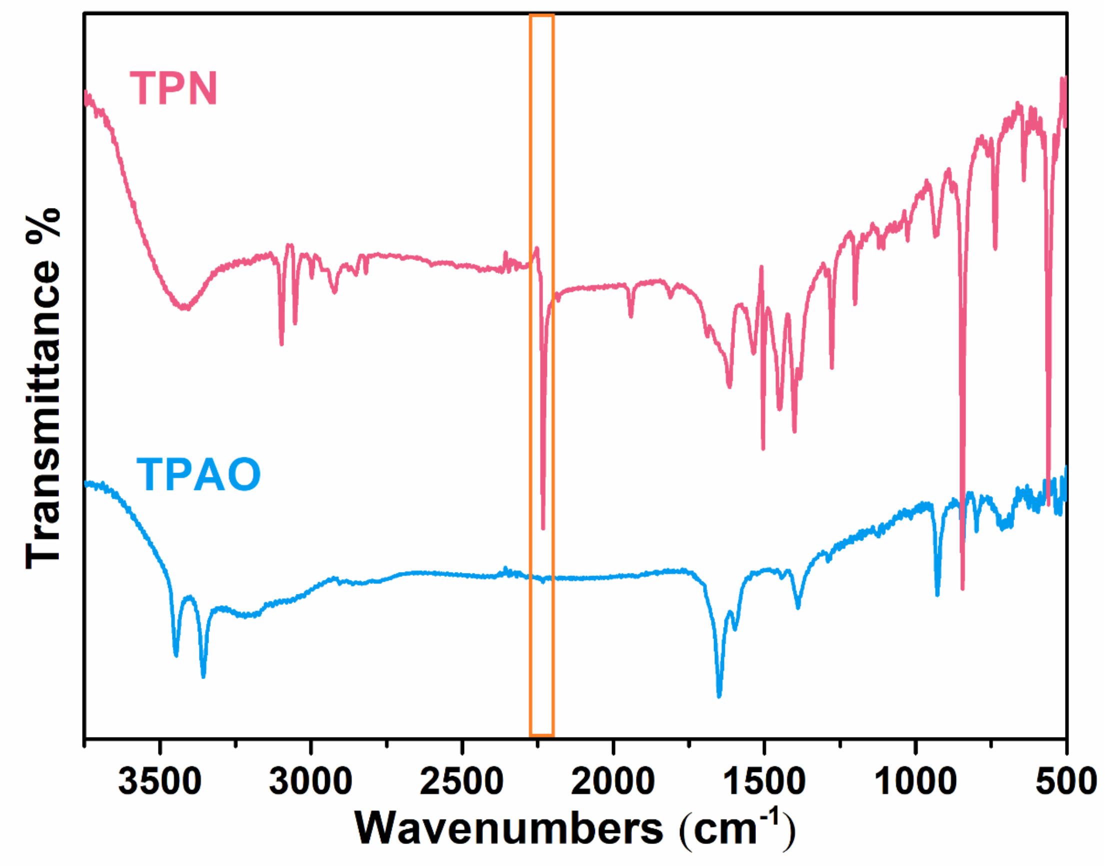

Supplementary Figure 1 The FTIR spectra of TPN and TPAO.

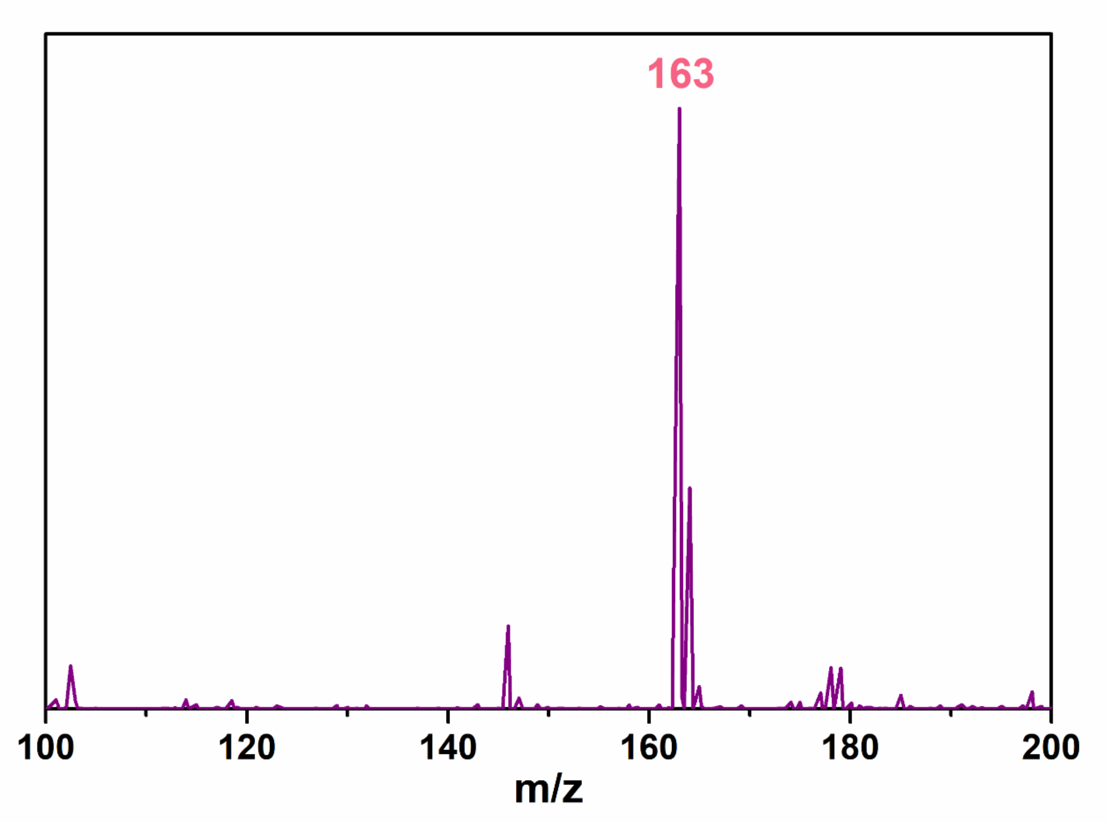

Supplementary Figure 2 The MS spectrum of XHOF-TAQ. MS (m/z): [M]+ calcd. for C8H12N4, 164.216; found, 163.251

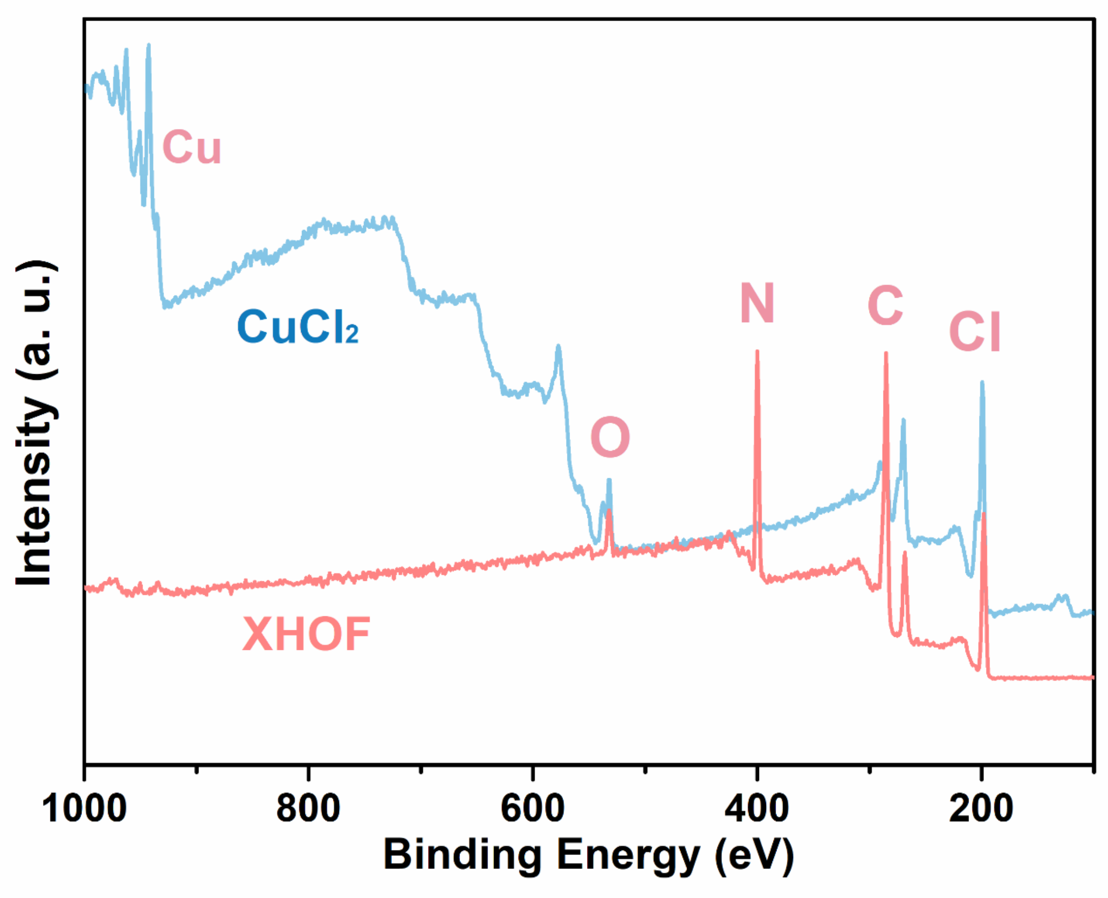

Supplementary Figure 3 The XPS spectra of $CuCl_2$ and XHOF-TAQ.

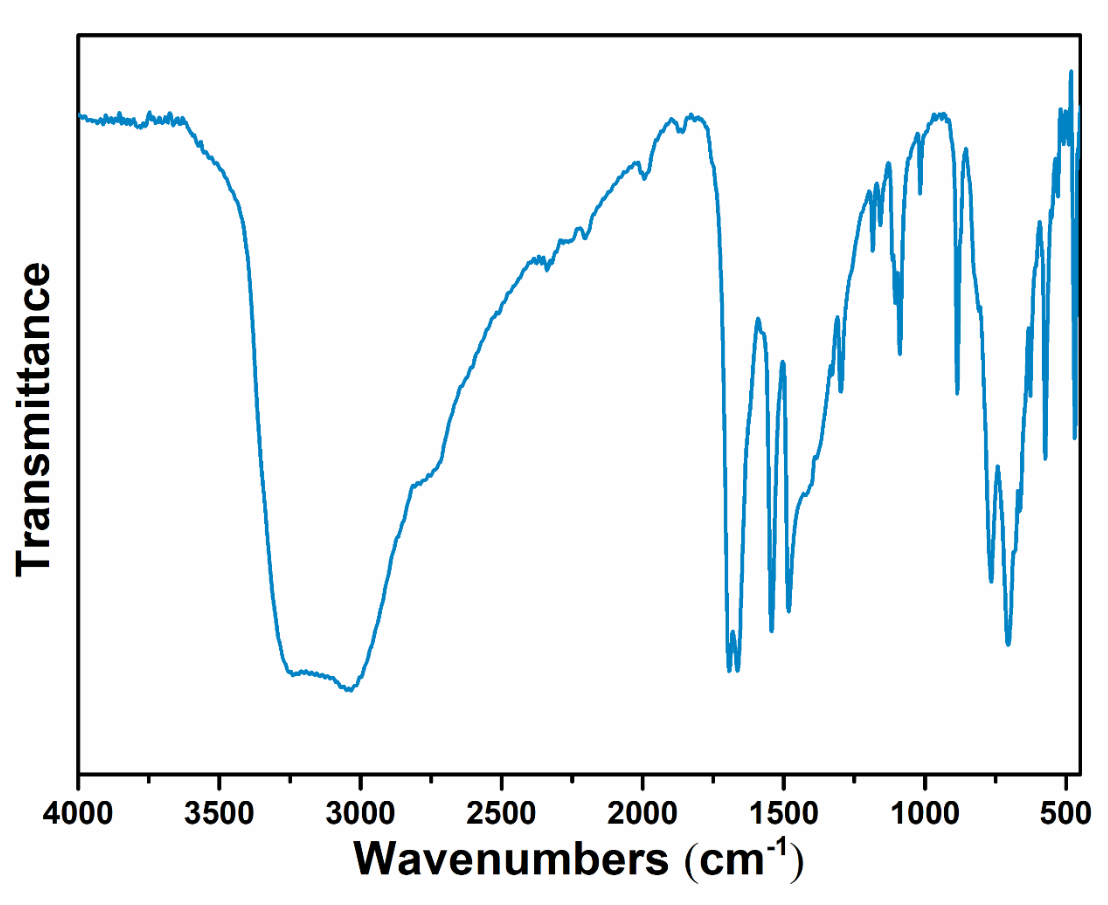

Supplementary Figure 4 The FTIR spectrum of XHOF-TAQ.

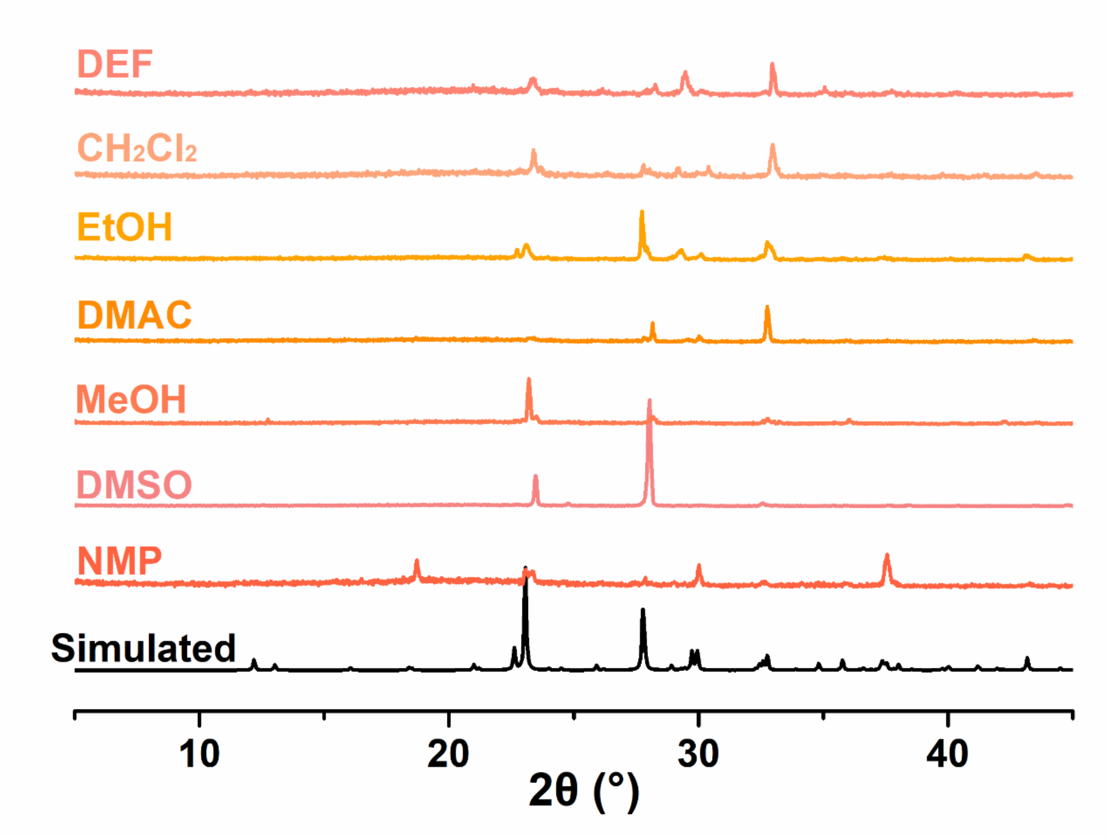

Supplementary Figure 5 The PXRD patterns of XHOF-TAQ in different solvents.

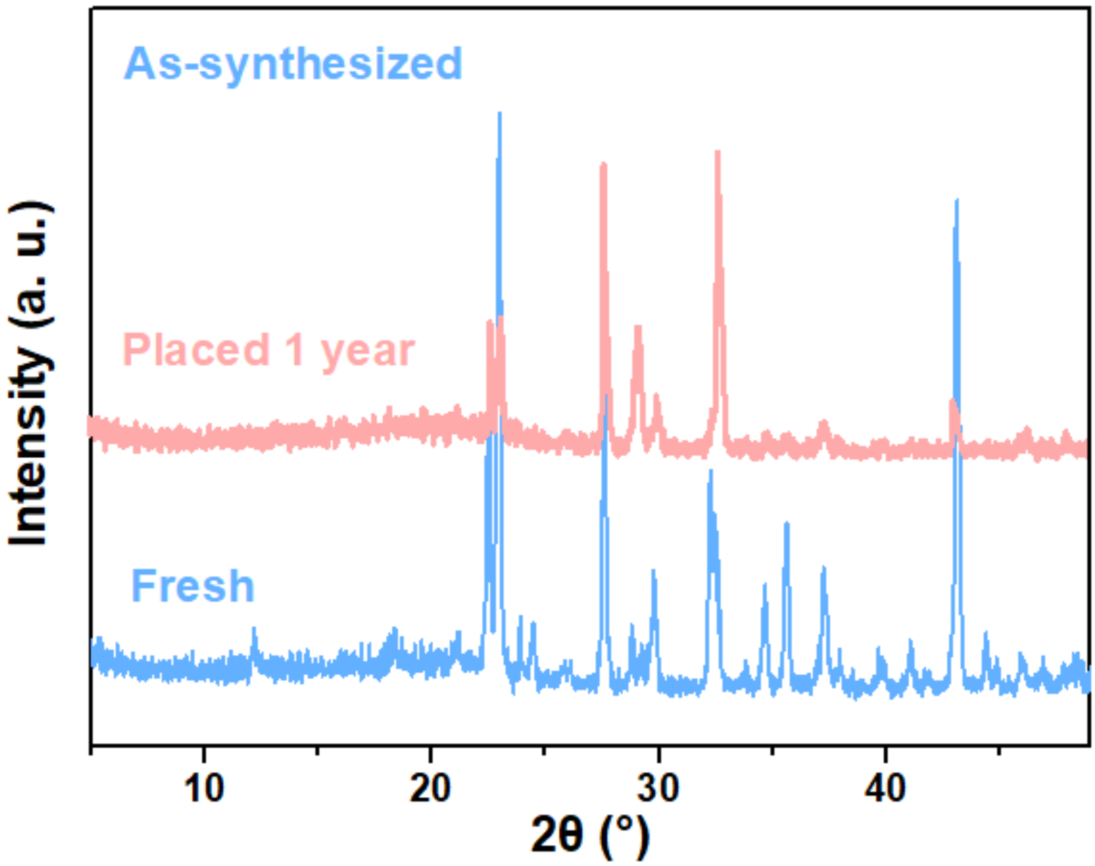

Supplementary Figure 6 PXRD pattern of the as-synthesized XHOF-TAQ and the XHOF-TAQ after being placed in air for one year.

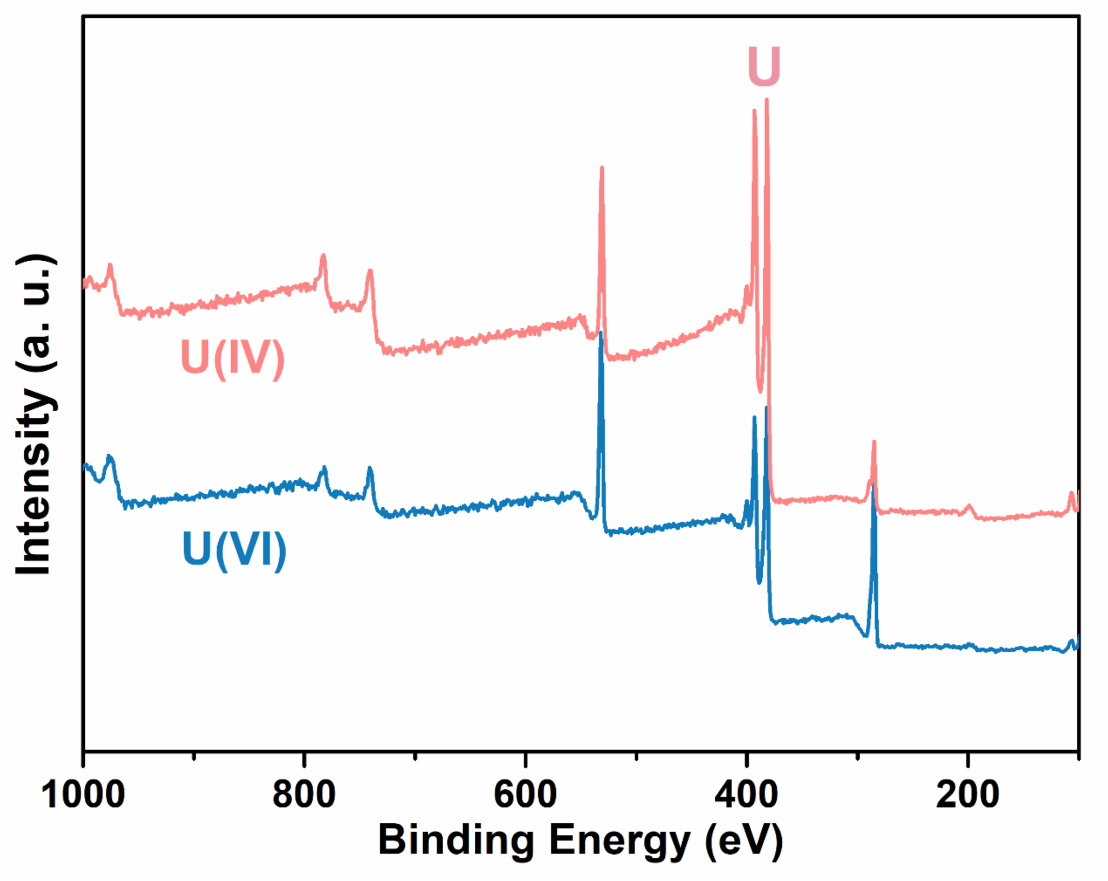

Supplementary Figure 7 The XPS spectra of before and after the reaction of uranium.

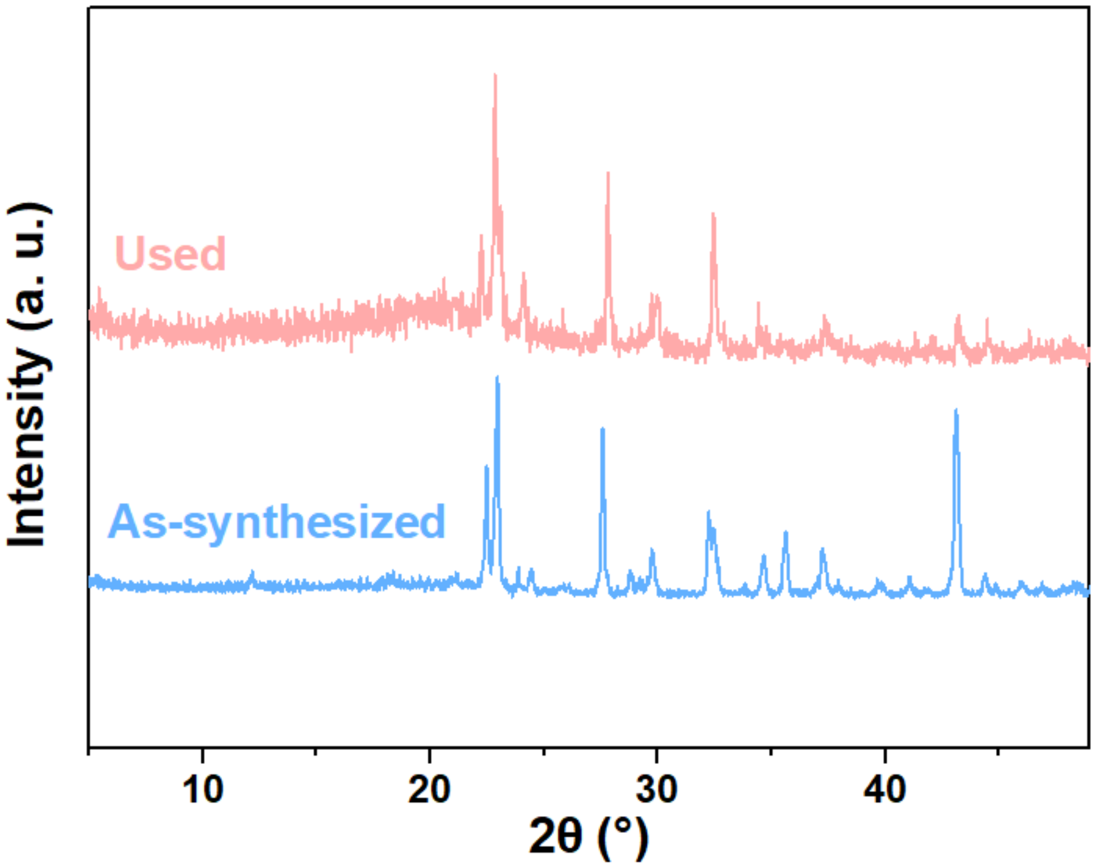

Supplementary Figure 8 PXRD pattern of **XHOF-TAQ** after being used for uranium photoreduction.

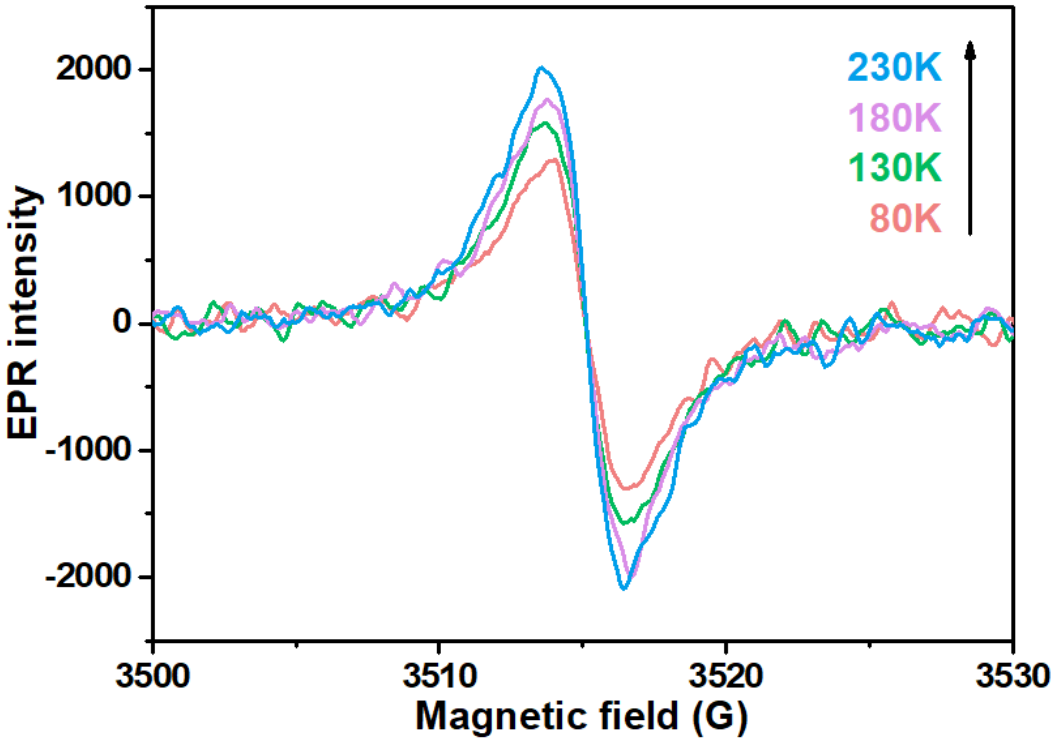

Supplementary Figure 9 The variable temperature EPR of the XHOF-TAQ.

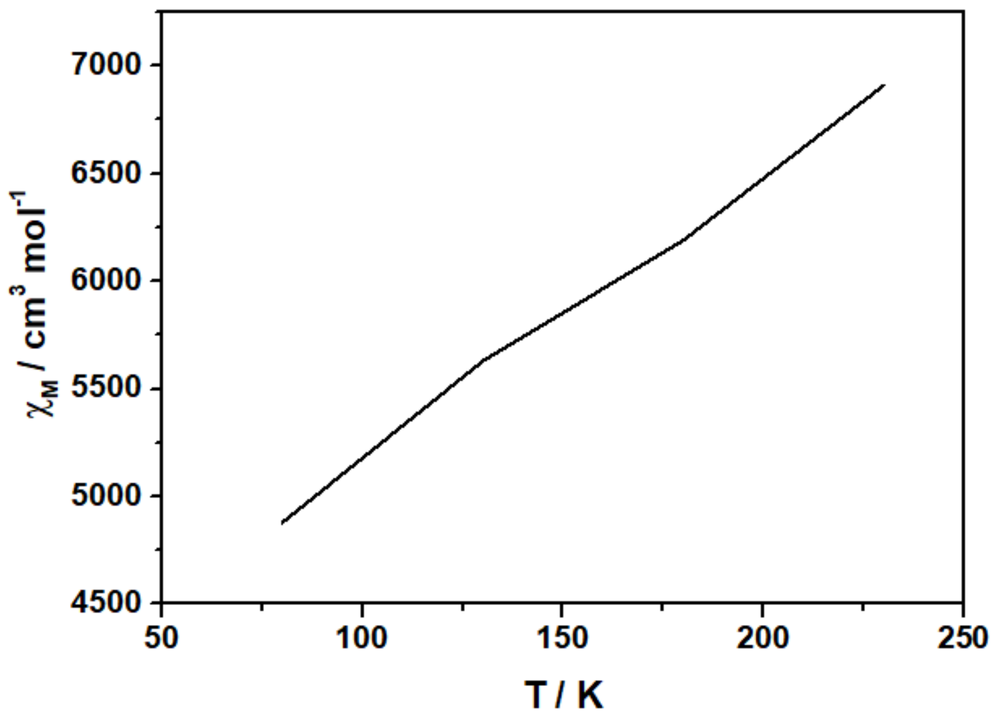

Supplementary Figure 10 Temperature dependence of χ and χ_M curve for the powder XHOF-TAQ.

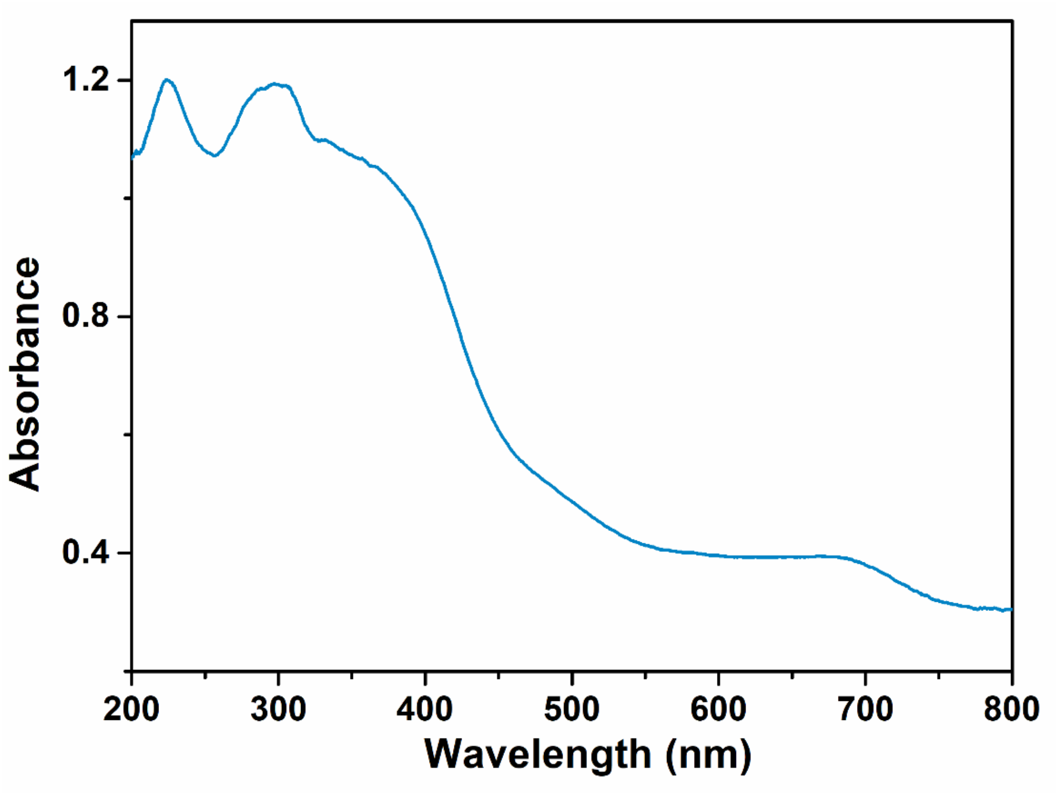

Supplementary Figure 11 The DRS spectrum of the XHOF-TAQ.

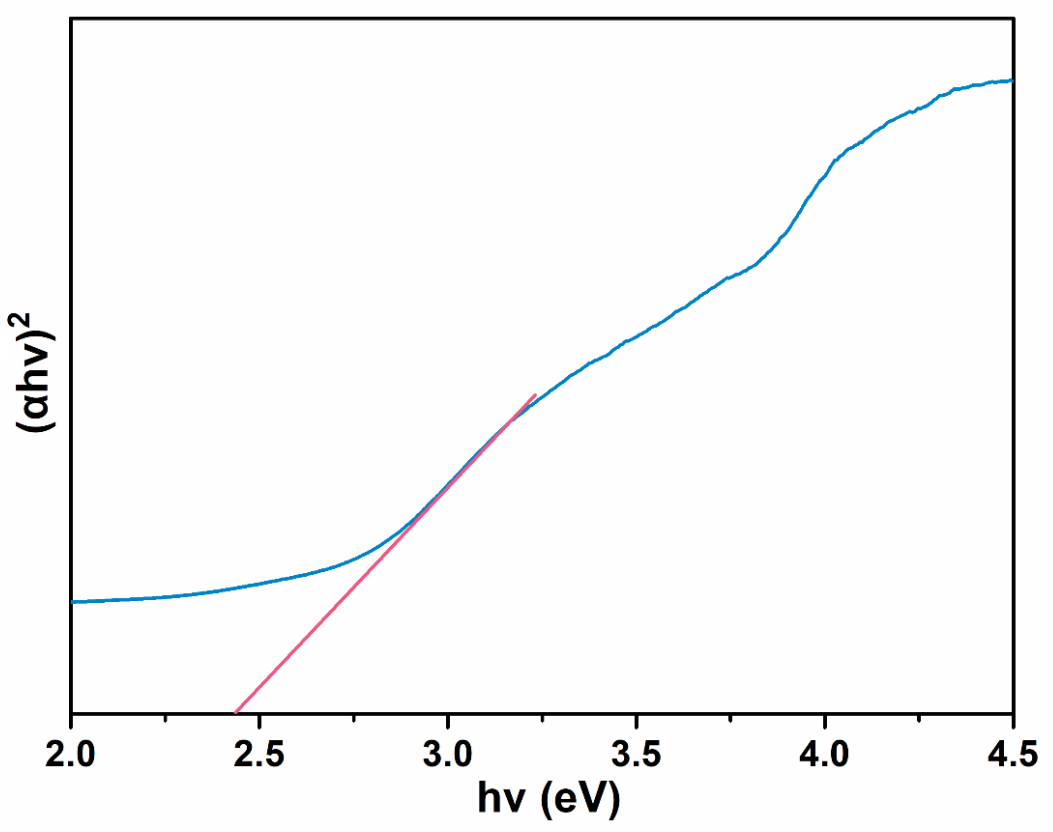

Supplementary Figure 12 The DRS spectrum was converted to the TAUC plot. The band gap (*E*g) of XHOF-TAQ is estimated to be 2.43 eV.

Supplementary Table

Supplementary Table 1 Comparison of uranium photoreduction performance with other available materials.

Catalysts	Dose g L^{-1}	Qe mg g^{-1}	Efficiency	Equilibrium time	Rate mg g^{-1} min^{-1}	Ref.
**XHOF-TAQ**	**0.05**	**1708**	**85%**	**50 min**	**34.16**	**This work**
DI-SNZVI	0.05	427.9	/	3 h	2.38	1
MOF@sponge	0.01	744.6	/	300 min	2.48	2
BP@CNF-MOF	0.01	800	98%	12 h	0.11	3
DC-PAO	0.01	421	/	45 h	0.16	4
AF COF	/	450	99.2%	50 min	9.00	5
TiO_2/Fe_3O_4	0.38	252	/	85 min	2.96	6
MOF SCU-19	0.50	500	/	50 h	0.17	7
Ti_3C_2/CdS	0.20	242.5	97%	40 min	6.06	8
TiO_2/CPAN-AO	0.20	2380	/	5 h	0.79	9
TiO_2 suspension	2.00	/	98.6%	30 h	/	10
DHBD-TMT	0.125	2640.8	99%	180 min	14.67	11
$ZnFe_2O_4$	0.20	245	98%	60 min	4.08	12
pTTT-Ben	1.00	4710	78%	4 h	19.63	13
CNBr	0.50	80	95%	20 min	4.00	14
ECUT-SO	0.50	1780	97.8%	60 min	29.67	15
Tp-TMT	0.125	2362.4	/	300 min	7.87	16
CdSTe-EDA	0.25	836	97.4%	70 min	11.94	17
CdS/g-C_3N_4	1.00	22.7	99%	20 min	1.14	18
BP-PAO	0.01	1000	80.39 %	32 h	0.52	19
TiO_2	0.60	3010	98%	300 min	10.03	20
g-CNNs	1.00	/	90%	2 h	/	21
PN-PCN-222	0.50	800	99%	1200 min	0.67	22
g-C_3N_4/TiO_2	0.40	25	99%	25 min	1.00	23
MoS_2/P-g-C_3N_4	1.00	90	99%	40 min	2.25	24
g-C_3N_4 MCN	0.50	47	99%	20 min	2.35	25
CMPs PTrSO-2	0.50	99.5	99.5%	120 min	0.83	26
CCN-24 g-C_3N_4	0.60	40	100%	50 min	0.80	27
$C_3N(5)$/RGO	0.20	50	94.9%	60 min	0.83	28
Sn-In_2S_3	0.15	/	95%	40 min	/	29
CuS/TNTAs	0.40	115.75	92.6 %	180 min	0.64	30

Te@O-SnS_2	0.25	704.8	97.3%	60 min	11.75	31
Ti_3C_2/$SrTiO_3$	0.34	115.5	77%	180 min	0.64	32
GA-200	0.40	1050	98%	180 min	5.83	33
PyB-SO_3H	0.20	1989	90%	60 min	33.15	34
mGO/g-C_3N_4	0.17	2880.6	96.02%	24 h	2.00	35
MoS_2/g-C_3N_4	1.00	33.2	83%	75 min	0.44	36
$WO_{2.78}$	0.25	507.2	95.6%	120 min	4.23	37
CN550 g-C_3N_4	0.20	1057	99%	1400 min	0.76	38
PFB/CN	0.50	200	99%	100 min	2.00	39
ipCN g-C_3N_4	1.00	/	98%	20 min	/	40
ZnS@/g-C_3N_4 ZSGCN-5	0.20	250	99%	160 min	1.56	41
rGO KTG	0.10	521.6	92.07%	600 min	0.87	42
ZIF-8/g-C_3N_4	0.10	100	98%	30 min	3.33	43
COF DBD-BTTH	0.01	400	/	6 h	1.11	44
H-VO_2	0.25	32	95.4%	90 min	0.36	45
Ag/ZIF-8	0.25	433.6	85.8%	20 min	21.68	46
2-PrOH	1.00	59	100%	60 min	0.98	47
BiOBr@COF	0.34	80	91%	540 min	0.15	48
Ag-SnS_2@$InVO_4$	0.25	120	97.8%	60 min	2.00	49
BCN-80	0.50	800	97.4%	1.5 h	8.89	50
g-C_3N_4/TiO_2	0.25	64	80%	250 min	0.26	51
MoSx/RGO	0.50	15	91.6%	60 min	0.25	52

Supplementary Table 2. The crystallographic data for XHOF-TAQ.

	XHOF-TAQ
Empirical formula	$C_8H_{12}N_4Cl_2$
Formula weight	235.12
Crystal system	monoclinic
Space group	*C2/c*
Unit cell dimensions	
a (Å)	12.9780(7)
b (Å)	9.6382(6)
c (Å)	9.9471(6)
α (°)	90.00
β (°)	121.812(3)
γ (°)	90.00
V ($Å^3$)	1057.32 (11)
Z	4
Dcalc (g cm^{-3})	1.477
F (000)	488.0
Crystal size (mm)	2×1.5×1
Melting point	~245 °C
Limiting indices	$-15 \le h \le 16$
	$-12 \le k \le 11$
	$-12 \le l \le 12$
Reflection collected	4229
Independent reflections	1061
Data/restraints/parameters	1061/0/64
Goodness-of-fit on F^2	1.091
R (reflections)	0.0570
wR2 (reflections)	0.1641

Supplementary References

1 Pang, H. W. *et al.* Adsorptive and reductive removal of U(VI) by Dictyophora indusiate-derived biochar supported sulfide NZVI from wastewater. *Chem. Eng. J.* **366**, 368-377 (2019).

2 Liu, T. *et al.* Photothermal enhancement of uranium capture from seawater by monolithic MOF-bonded carbon sponge. *Chem. Eng. J.* **412**, 128700 (2021).

3 Chen, M. W. *et al.* Photoinduced Enhancement of Uranium Extraction from Seawater by MOF/Black Phosphorus Quantum Dots Heterojunction Anchored on Cellulose Nanofiber Aerogel. *Adv. Funct. Mater.* **31**, 2100106 (2021).

4 Wang, N. *et al.* Accelerated Chemical Thermodynamics of Uranium Extraction from Seawater by Plant-Mimetic Transpiration. *Adv. Sci.* **8**, 2102250 (2021).

5 Wu, Y. D., Cui, W. R., Zhang, C. R., Liang, R. P. & Qiu, J. D. Regenerable, anti-biofouling covalent organic frameworks for monitoring and extraction of uranium from seawater. *Environ. Chem. Lett.* **19**, 1847-1856 (2021).

6 Li, Z. J. *et al.* Enhanced Photocatalytic Removal of Uranium(VI) from Aqueous Solution by Magnetic TiO2/Fe3O4 and Its Graphene Composite. *Environ. Sci. Technol.* **51**, 5666-5674 (2017).

7 Zhang, H. L. *et al.* Three Mechanisms in One Material: Uranium Capture by a Polyoxo-metalate-Organic Framework through Combined Complexation, Chemical Reduction, and Photocatalytic Reduction. *Angew. Chem. Int. Edit.* **58**, 16110-16114 (2019).

8 Liang, P. L. *et al.* Photocatalytic reduction of uranium(VI) under visible light

with 2D/ 1D Ti3C2/CdS. *Chem. Eng. J.* **420**, 129831 (2021).

9 Xu, Y. C. *et al.* Surface hybridization of pi-conjugate structure cyclized polyacrylonitrile and radial microsphere shaped TiO2 for reducing U(VI) to U(IV). *J. Hazard. Mater.* **416**, 125812 (2021).

10 Liu, M. X. *et al.* Characteristics and mechanism of uranium photocatalytic removal enhanced by chelating hole scavenger citric acid in a TiO2 suspension system. *J. Radioanal. Nucl. Ch.* **319**, 147-158 (2019).

11 Cui, W. R. *et al.* Rational design of covalent organic frameworks as a groundbreaking uranium capture platform through three synergistic mechanisms. *Appl. Catal. B-Environ.* **294**, 120250 (2021).

12 Liang, P. L. *et al.* Photocatalytic reduction of uranium(VI) by magnetic ZnFe2O4 under visible light. *Appl. Catal. B-Environ.* **267**, 118688 (2020).

13 Chen, B. *et al.* Visible light driven photocatalytic removal of uranium(VI) in strongly acidic solution. *J. Hazard. Mater*. **21**, 127851 (2021).

14 Xue, J. M., Wang, B., Li, Z. Q., Xie, Z. B. & Le, Z. G. Bromine doped g-C3N4 with enhanced photocatalytic reduction in U(VI). *Res. Chem. Intermediat.*, doi:10.1007/s11164-021-04568-7 (2021).

15 Yu, F. T. *et al.* Tunable perylene-based donor-acceptor conjugated microporous polymer to significantly enhance photocatalytic uranium extraction from seawater. *Chem. Eng. J.* **412**, 127558 (2021).

16 Xu, R. H. *et al.* Vinylene-linked covalent organic frameworks with enhanced uranium adsorption through three synergistic mechanisms. *Chem. Eng. J.* **419**,

129550 (2021).

17 Dong, C. X. *et al.* Efficient Photocatalytic Extraction of Uranium over Ethylenediamine Capped Cadmium Sulfide Telluride Nanobelts. *Acs. Appl. Mater. Inter.* **13**, 11968-11976 (2021).

18 Li, P. *et al.* Ultrafast recovery of aqueous uranium: Photocatalytic U(VI) reduction over CdS/g-C3N4. *Chem. Eng. J.* **425**, 131552 (2021).

19 Yuan, Y. H. *et al.* Photoinduced Multiple Effects to Enhance Uranium Extraction from Natural Seawater by Black Phosphorus Nanosheets. *Angew. Chem. Int. Edit.* **59**, 1220-1227 (2020).

20 Wang, Y. *et al.* Efficient recovery of uranium from saline lake brine through photocatalytic reduction. *J. Mol. Liq.* **308**, 113007 (2020).

21 Liu, C. *et al.* Study on photocatalytic performance of hexagonal SnS2/g-C3N4 nanosheets and its application to reduce U (VI) in sunlight. *Appl. Surf. Sci.* **537**, 147754 (2021).

22 Hui, L. *et al.* Powerful uranium extraction strategy with combined ligand complexation and photocatalytic reduction by postsynthetically modified photoactive metal-organic frameworks. *Appl. Catal. B-Environ.* **254**, 47-54 (2019).

23 Liu, Y. L., Wu, S. S., Liu, J., Xie, S. B. & Liu, Y. J. Synthesis of g-C3N4/TiO2 nanostructures for enhanced photocatalytic reduction of U(vi) in water. *Rsc Adv.* **11**, 4810-4817 (2021).

24 Li, Z. F. *et al.* Synthesis of MoS2/P-g-C3N4 nanocomposites with enhanced

visible-light photocatalytic activity for the removal of uranium (VI). *J. Solid. State. Chem.* **302**, 122305 (2021).

25 Wang, J. J. *et al.* Tunable mesoporous g-C3N4 nanosheets as a metal-free catalyst for enhanced visible-light-driven photocatalytic reduction of U(VI). *Chem. Eng. J.* **383**, 123193 (2020).

26 Yu, F. T. *et al.* Novel donor-acceptor-acceptor ternary conjugated microporous polymers with boosting forward charge separation and suppressing backward charge recombination for photocatalytic reduction of uranium (VI). *Appl. Catal. B-Environ.* **301**, 120819 (2022).

27 Li, P. *et al.* Carboxyl groups on g-C3N4 for boosting the photocatalytic U(VI) reduction in the presence of carbonates. *Chem. Eng. J.* **414**, 128810 (2021).

28 Wu, L. Z. *et al.* Three-dimensional C3N(5)/RGO aerogels with enhanced visible-light response and electron-hole separation efficiency for photocatalytic uranium reduction. *Chem. Eng. J.* **427** 131773 (2022).

29 Feng, J. N. *et al.* Photocatalytic reduction of Uranium(VI) under visible light with Sn-doped In2S3 microspheres. *Chemosphere* **212**, 114-123 (2018).

30 Li, Z. F. *et al.* CuS/TiO2 nanotube arrays heterojunction for the photoreduction of uranium (VI). *J. Solid. State. Chem.* **303**, 122499 (2021).

31 Lei, J. *et al.* Tellurium nanowires wrapped by surface oxidized tin disulfide nanosheets achieves efficient photocatalytic reduction of U(VI). *Chem. Eng. J.* **426**, 130756 (2021).

32 Deng, H. *et al.* Nanolayered Ti3C2 and SrTiO3 Composites for Photocatalytic

Reduction and Removal of Uranium(VI). *Acs Appl. Nano. Mater.* **2**, 2283-2294, (2019).

33 Wang, Z. *et al.* Graphene aerogel for photocatalysis-assist uranium elimination under visible light and air atmosphere. *Chem. Eng. J.* **402**, 126256 (2020).

34 Yu, F. T., Song, F. R., Wang, R. Z., Xu, M. & Luo, F. Sulfonated perylene-based conjugated microporous polymer as a high-performance adsorbent for photo-enhanced uranium extraction from seawater. *Polym. Chem-Uk.* **12**, 867-875 (2021).

35 Dai, Z. R., Sun, Y. S., Zhang, H., Ding, D. X. & Li, L. Photocatalytic reduction of U(VI) in wastewater by mGO/g-C3N4 nanocomposite under visible LED light irradiation. *Chemosphere* **254**, 126671 (2020).

36 Zhang, Z. B. *et al.* Synthesis of flower-like MoS 2 /g-C 3 N 4 nanosheet heterojunctions with enhanced photocatalytic reduction activity of uranium(VI). *Appl. Surf. Sci.* **520**, 146352 (2020).

37 Lei, J. *et al.* Enhanced photoreduction of U(VI) on WO3 nanosheets by oxygen defect engineering. *Chem. Eng. J.* **416**, 129164 (2021).

38 Liu, S. *et al.* Sunlight-induced uranium extraction with triazine-based carbon nitride as both photocatalyst and adsorbent. *Appl. Catal. B-Environ.* **282**, 119523 (2021).

39 Yu, F. T. *et al.* Heteroatom engineering of polymeric carbon nitride heterojunctions for boosting photocatalytic reduction of hexavalent uranium. *Mol. Syst. Des. Eng.* **5**, 882-889 (2020).

40 Le, Z. G. *et al.* Self-cleaning isotype g-C3N4 heterojunction for efficient photocatalytic reduction of hexavalent uranium under visible light. *Environmental Pollution* **260**, 114070 (2020).

41 Wu, F. *et al.* The enhanced photocatalytic reduction of uranium(VI) by ZnS@g-C3N4 heterojunctions under sunlight. *J. Radioanal. Nucl. Ch.* **329**, 1125-1133 (2021).

42 Zhang, C. R. *et al.* rGO-based covalent organic framework hydrogel for synergistically enhance uranium capture capacity through photothermal desalination. *Chem. Eng. J.* **428**, 131178 (2022).

43 Qiu, M. Q., Liu, Z. X., Wang, S. Q. & Hu, B. W. The photocatalytic reduction of U(VI) into U(IV) by ZIF-8/g-C3N4 composites at visible light. *Environ. Res.* **196**, 110349 (2021).

44 Cui, W. R., Zhang, C. R., Liang, R. P., Liu, J. & Qiu, J. D. Covalent Organic Framework Sponges for Efficient Solar Desalination and Selective Uranium Recovery. *ACS Appl. Mater. Interfaces.* **13**, 31561-31568 (2021).

45 Liu, H. H. *et al.* Hydrogen-incorporated vanadium dioxide nanosheets enable efficient uranium confinement and photoreduction. *Nano. Res.*, doi:10.1007/s12274-021-3916-8 (2021).

46 Jiang, P. Y. *et al.* Encapsulating Ag nanoparticles into ZIF-8 as an efficient strategy to boost uranium photoreduction without sacrificial agents. *J. Mater. Chem. A.* **9**, 9809-9814 (2021).

47 Salomone, V. N., Meichtry, J. M., Zampieri, G. & Litter, M. I. New insights in

the heterogeneous photocatalytic removal of U(VI) in aqueous solution in the presence of 2-propanol. *Chem. Eng. J.* **261**, 27-35 (2015).

48 Zhong, X., Liu, Y. X., Wang, S., Zhu, Y. L. & Hu, B. W. In-situ growth of COF on BiOBr 2D material with excellent visible-light-responsive activity for U(VI) photocatalytic reduction. *Sep. Purif. Technol.* **279**, 119627 (2021).

49 He, S., Yang, Z. Q., Cui, X. D., Zhang, X. Y. & Niu, X. J. Fabrication of the novel Ag-doped SnS2@InVO4 composite with high adsorption-photocatalysis for the removal of uranium (VI). *Chemosphere* **260**, 127548 (2020).

50 Wang, Y. *et al.* Carbon-doped boron nitride nanosheets with adjustable band structure for efficient photocatalytic U(VI) reduction under visible light. *Chem. Eng. J.* **410**, 128280 (2021).

51 Jiang, X. H. *et al.* Simultaneous photoreduction of Uranium(VI) and photooxidation of Arsenic (III) in aqueous solution over g-C3N4/TiO2 heterostructured catalysts under simulated sunlight irradiation. *Appl. Catal. B-Environ.* **228**, 29-38 (2018).

52 Chen, T. *et al.* Harmonizing the energy band between adsorbent and semiconductor enables efficient uranium extraction. *Chem. Eng. J.* **420**, 127645 (2021).
